# Optimization of Thermal Weld Parameters for the Loading Neck of a Flexible Polyethylene Container

**DOI:** 10.3390/polym18212740

**Published:** 2026-11-04

**Authors:** Bekbossyn Kassymbayev, Adilkhan Niyazbayev, Issa Tailer, Yelaman Zhumagaliyev, Baydaulet Urmashev, Aliya Borsikbayeva, Yerzhigit Saparbayev, Marat Khazimov

**Affiliations:** 1 Faculty of Engineering and Technology, Kazakh National Agrarian Research University, Almaty 050010, Kazakhstan; bekbossyn.kassymbayev@kaznaru.edu.kz (B.K.); adil77@mail.ru (A.N.); igaliyev.issa@kaznaru.edu.kz (I.T.); zhumagaliyev.yelaman@kaznaru.edu.kz (Y.Z.); a.borsikbaeva@kbtu.kz (A.B.); yerzhigit.saparbayev@gmail.com (Y.S.); 2 School of Transport Engineering and Logistics Named After M. Tynyshpayev, Kazakh National Technical Research University Named After K.I. Satbayev, Almaty 050013, Kazakhstan; 3 Faculty of Information Technology, Al-Farabi Kazakh National University, Almaty 050040, Kazakhstan; baydaulet.urmashev@gmail.com; 4 Institute of Energy and Green Technologies, Almaty University of Power Engineering and Telecommunications Named After Gumarbek Daukeyev, Almaty 050013, Kazakhstan; 5 School of Applied Mathematics, Kazakh British Technical University, Almaty 050005, Kazakhstan

**Keywords:** vacuum compaction, flexible container, thermal weld

## Abstract

This study presents an experimental and numerical investigation of the sealing performance of welded joints in flexible polyethylene containers subjected to external pressure during vacuum compaction of forage material. The objective was to improve the reliability of welded seams under vacuum gauge pressures of up to 75 kPa. A three-dimensional transient heat-conduction model was developed to investigate the thermal behavior of polyethylene film during heat sealing. The governing heat-conduction equation was solved using ANSYS Fluent based on the finite volume method, employing a pressure-based transient solver and first-order implicit time discretization. Temperature distributions and their temporal evolution, T(t), were determined at selected locations within the film. The strength of welded joints in thin polyethylene films subjected to pressure loading of up to 75 kPa was experimentally evaluated as a function of weld-seam width and clamping force at a selected heat-sealing temperature of 175 °C. High-density polyethylene film (PE-180) with a thickness of 200 μm was used to carry out research specimens measuring 100 × 100 mm. The experimental results were analyzed using a design-of-experiments approach, and a regression equation was developed to describe the dependence of welded-joint strength on seam width and clamping force. The combined numerical and experimental findings provide a basis for selecting appropriate heat-sealing parameters to enhance the reliability of welded seams at the neck of flexible polyethylene containers subjected to vacuum-induced loading.

## 1. Introduction

A stable daily supply of feed is essential for the sustainable development of livestock production. This challenge is particularly pronounced in arid regions, where droughts and interannual variations in crop yields limit feed availability [1]. An increase of 2 °C in mean annual temperature adversely affects pastures and livestock production in arid and semi-arid regions, whereas its effects may be positive in humid temperate regions [2]. Kazakhstan is a major livestock-producing country, with a substantial proportion of its territory occupied by steppes and deserts [3]. Consequently, the ensiling of succulent forage crops, particularly maize, is an important approach to establishing feed reserves for winter and drought periods [4]. Maize silage is widely used in livestock production because of its nutritional value and feeding efficiency. However, challenges remain in optimizing silage quality [5] and controlling pathogenic microorganisms. Inadequate ensiling conditions, particularly oxygen ingress and insufficient acidification, can promote the growth of *Listeria monocytogenes*, *Clostridium botulinum*, and *Bacillus cereus* [6]. Furthermore, adapting forage production and preservation technologies to climate change remains an important challenge [7].

Successful silage preservation depends on three essential conditions: adequate acidification of the forage mass, oxygen exclusion, and moisture control. Among these factors, preventing oxygen ingress throughout the storage period is particularly important, especially when storage extends to 15 months under variable environmental conditions [8]. Silage quality is strongly influenced by the rate and completeness of oxygen removal and the effectiveness of maintaining anaerobic conditions. Oxygen penetration promotes the development of yeasts, molds, and other aerobic microorganisms, resulting in dry matter losses and reduced aerobic stability [9]. Packing density is a key physical parameter influencing these processes because it determines the porosity of the forage mass and the rate of oxygen penetration [10]. Vacuum-assisted compaction is considered a promising approach for controlling packing density and improving silage preservation [11,12]. Nevertheless, reliable heat sealing of flexible containers used for vacuum compaction remains an important technological challenge.

In conventional bunker silos, oxygen ingress before opening is primarily associated with three factors: the intrinsic oxygen permeability of the covering film, mechanical damage occurring during film manufacture or installation, and improper overlapping or fixation of the film, which may allow movement of the cover [8,13]. In flexible vacuum containers with an otherwise continuous and hermetically sealed envelope, the welded seam at the container neck represents a particularly critical location for maintaining airtightness. This seam is formed under field conditions, where environmental and operating parameters may vary. It must withstand mechanical stresses arising from vacuum-induced pressure differences and the forces exerted by the compacted forage mass during handling, transportation, and storage. Failure of the welded joint may result in loss of vacuum, oxygen ingress, and subsequent deterioration of the stored forage [14].

The reliability of heat-sealed joints in polyethylene films is primarily governed by sealing temperature, applied pressure, and dwell time [15]. During heat sealing, the contacting film surfaces must reach a temperature sufficient to enable polymer-chain mobility and interdiffusion across the interface, thereby forming a continuous welded joint. The relationship between sealing temperature and joint strength is generally nonlinear and may exhibit an S-shaped response. Initially, a threshold temperature must be reached to form a load-bearing joint, followed by an increase in strength and eventually a plateau beyond which additional heating provides little or no improvement [16]. Insufficient heating results in incomplete fusion and reduced joint strength. Conversely, excessive heating may cause local thinning near the seam edges and deterioration of mechanical performance. Importantly, weld formation and strength depend not only on the heater temperature but also on the actual temperature at the film–film interface [17]. This interfacial temperature is influenced by heat transfer to the supporting surfaces, pressing elements, and surrounding materials [18,19]. Previous molecular-level investigations have also demonstrated that temperature equalization between polymer films initially at different temperatures may be asymmetric, influencing the onset of macromolecular interdiffusion across the interface. Under practical conditions, the effective heating efficiency of press-type film-sealing equipment is typically within the range of 0.3–0.6 [20].

Numerical modeling provides an effective approach for investigating the complex thermal processes involved in polymer welding. It enables the analysis of transient heat transfer, prediction of temperature distributions, and evaluation of thermal conditions affecting weld formation [21,22,23]. Previous investigations have developed thermal models of welded regions in multilayer polymer films using the finite difference method. These studies demonstrated that temperature distributions may differ substantially from those predicted by one-dimensional steady-state approximations because of thermal contact resistance between film layers. Subsequent investigations also incorporated rheological viscosity models to describe molten polymer flow between welding elements [24]. Numerical and analytical approaches make it possible to investigate relationships between process parameters and welding outcomes while reducing the need for extensive and costly experimental testing [25,26]. Computational fluid dynamics has also been applied to simulate convective and radiative heat transfer, transient temperature distributions, and molten-layer thickness during hot-gas welding of polypropylene, with reported differences between predicted and experimentally measured surface temperatures of less than 10%.

Transient heat transfer associated with moving heat sources has also been investigated for various thermoplastic welding processes. In induction welding of thermoplastic composites, a three-dimensional transient finite element model incorporating the movement of an induction coil along the weld line at a constant velocity was used to predict temperature distributions, melting, and crystallization at the joint interface. The model accounted for convective heat exchange with the surrounding air and conductive heat transfer through the pressing roller [27]. A similar approach was employed for continuous resistance welding of thermoplastic composites, where a moving electrical current source generates heat as the welding process progresses along the joining surfaces [28]. These investigations demonstrate the importance of transient heat-transfer modeling for extended linear welds produced by moving heat sources. This approach is also relevant to the self-propelled welding module considered in the present study, because the heating duration at each location along the seam depends on the travel speed of the welding device.

However, previous numerical investigations have primarily focused on thermoplastic materials with thicknesses exceeding 0.5 mm, whereas the mechanical reliability of welded joints in thin polyethylene films under vacuum-induced loading has received comparatively limited attention. In particular, the combined effects of weld-seam width and clamping force on joint strength require further investigation. Experimental determination of the temperature distribution through thin polymer films is challenging because of their small thickness and the transient nature of the heating process. Numerical modeling can therefore provide valuable information on the thermal conditions governing weld formation, while experimental testing can establish the relationships between controllable welding parameters and joint strength.

The objective of this study is to improve the reliability of welded joints at the neck of flexible polyethylene containers filled with haylage and subsequently subjected to vacuum compaction at vacuum gauge pressures of up to 75 kPa. The scientific novelty lies in combining a three-dimensional transient heat-conduction model of the heat-affected region in thin polyethylene film with an experimentally derived regression model describing welded-joint strength under vacuum-induced loading. This integrated approach provides a basis for determining appropriate heat-sealing parameters for flexible forage-storage containers. To achieve this objective, the following tasks were undertaken: (i) to develop a numerical model of transient heat transfer in the welding region based on the heat-conduction equation; (ii) to conduct experimental investigations of welded-joint formation in polyethylene film specimens using the numerical results to guide the selection of process conditions; (iii) to evaluate the ultimate strength of the welded joints experimentally; and (iv) to establish the ranges of welding parameters that provide maximum joint strength based on the experimental findings.

## 2. Materials and Methods

### 2.1. Mathematical Model and Numerical Simulation

The numerical simulation addresses the sealing process at the seams of a flexible polyethylene container used for the vacuum packaging of succulent forage (silage). In the contact zone with the self-propelled welding module, the film surface undergoes localized heating, followed by heat propagation into the material through conduction. To analyze the thermal effects of this process in the weld zone, a three-dimensional transient heat conduction problem is formulated for a solid body (the polyethylene film) subjected to a prescribed localized surface heat source.

The computational domain is a rectangular parallelepiped measuring 100 × 30 × 0.4 mm, representing a section of the polyethylene film. The heating zone was defined as a narrow strip 10 mm in width on the film surface, corresponding to the characteristic contact size of the welding tool with the material.

The three-dimensional heat conduction equation in Cartesian coordinates is written as:

(1) $$\frac{\partial T}{\partial t}=\alpha \left(\frac{\partial^{2}T}{\partial x^{2}}+\frac{\partial^{2}T}{\partial y^{2}}+\frac{\partial^{2}T}{\partial z^{2}}\right)$$

where *T* = *T*(*x*,*y*,*z*,*t*)—is temperature, *ρ*—is the material density, *C_p_*—is the specific heat capacity, *k*—is the thermal conductivity, $\alpha =\frac{k}{\rho C_{p}}$—is the thermal diffusivity.

The thermophysical properties of polyethylene (HDPE) used in the calculation for simulation are presented in (Table 1).

The initial condition corresponds to a uniform temperature distribution throughout the film volume, equal to the ambient temperature: *T*(*x*,*y*,*z*,0) = 298.15 K.

At the surface of the heating zone, a first-type (Dirichlet) boundary condition was prescribed, corresponding to the operating temperature of the welding element: *T_h_* = 450.15 K. The heating zone was applied on both the upper and lower surfaces of the film (two-sided heating).

A structured mesh was used to discretize the computational domain. The resulting mesh comprised 96,000 elements and 145,563 nodes.

A structured mesh was used to discretize the computational domain. The resulting mesh comprised 120,000 elements and 134,431 nodes.

The heat conduction equation was solved numerically in the ANSYS Fluent software (2020) package using the finite volume method. A pressure-based solver was employed in a transient formulation. Time discretization was performed using a first-order implicit scheme.

### 2.2. Experimental Study of the Strength of Weld Seams of Polyethylene Film

The experimental investigation was conducted to evaluate the effects of weld-seam width and clamping force on the resistance of polyethylene film joints to pressure-induced rupture. The rupture pressure was used as the response variable to characterize the performance of welded joints under the investigated loading conditions.

Polyethylene film PE-200 (Moscow, Russia, Webpack) (200 μm thick) was used as the material to be tested. Before testing, from film were prepared the samples with size 100 × 100 mm. The samples were subjected to a visual inspection in order to exclude mechanical damage, wrinkles, tears, contamination and other defects that could negatively influence the process of forming the weld and the results of subsequent tests.

**Figure 1:**
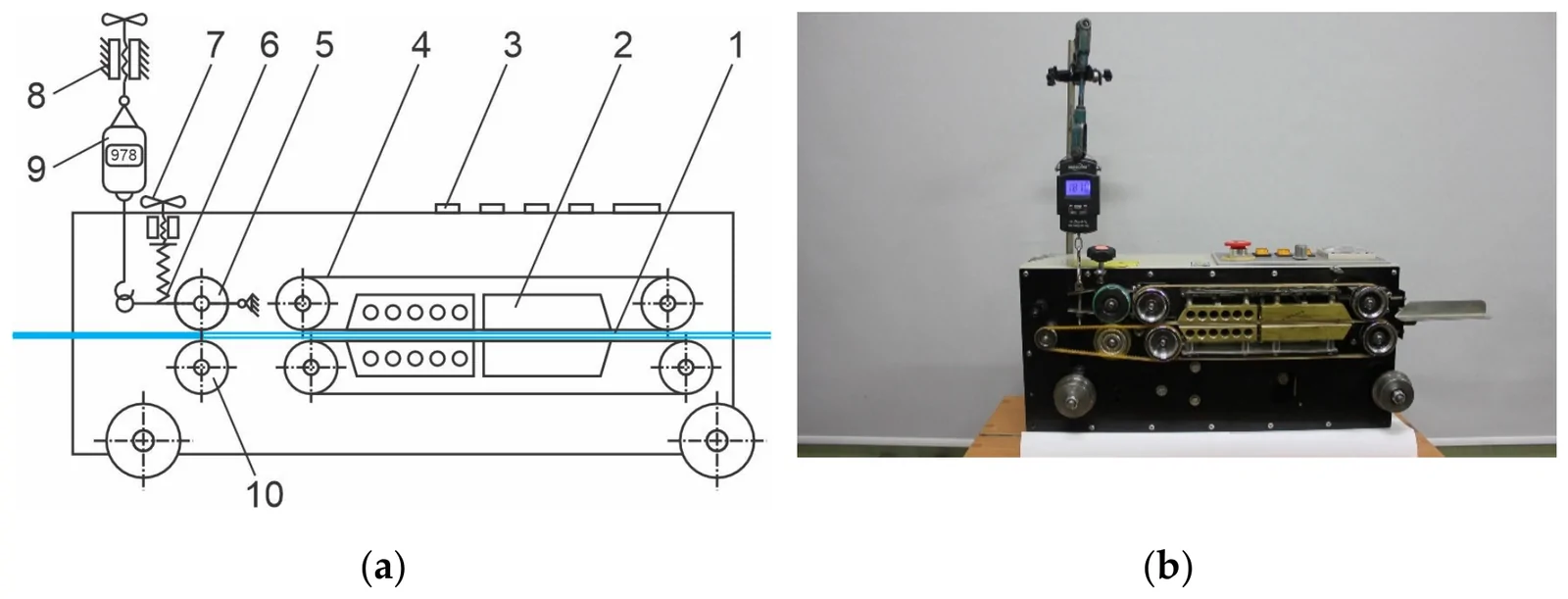
Diagram (**a**) and general view (**b**) of DBF-900W type film welding module: 1—polyethylene film; 2—heating element; 3—control panel; 4—Teflon tape with rollers; 5—movable push-roller; 6—backlash spring; 7—screw mechanism of pinch roller; 8—screw suspension mechanism; 9—digital load indicator; 10—lower push roller.

The samples were heat-sealed using a DBF-900W continuous welder with a power output of 0.65 kW (Figure 1). Formation of the welded compound occurred under successive effects of temperature and mechanical compression on welded layers of polyethylene film. During the passage of the sample film through the working area of the heater, the surface layers of polyethylene were heated, melted and joined under the applied force.

During experimental studies, the temperature of thermowelders was maintained at a constant 175 °C [18]. The fixed temperature value allowed us to exclude the influence of the temperature factor and to assess the impact of the studied parameters—weld width and compressive force—on the strength of the joint.

For quantitative control of the clamping force, the working part of the welding device was equipped with a measuring device (Eurecare EC-H05) with digital indication. The adjustment of forces was carried out by means of a screw mechanism, changing the positions of the clamping unit. The required force value was set immediately before each experiment and monitored by the readings of the measuring device.

**Figure 2:**
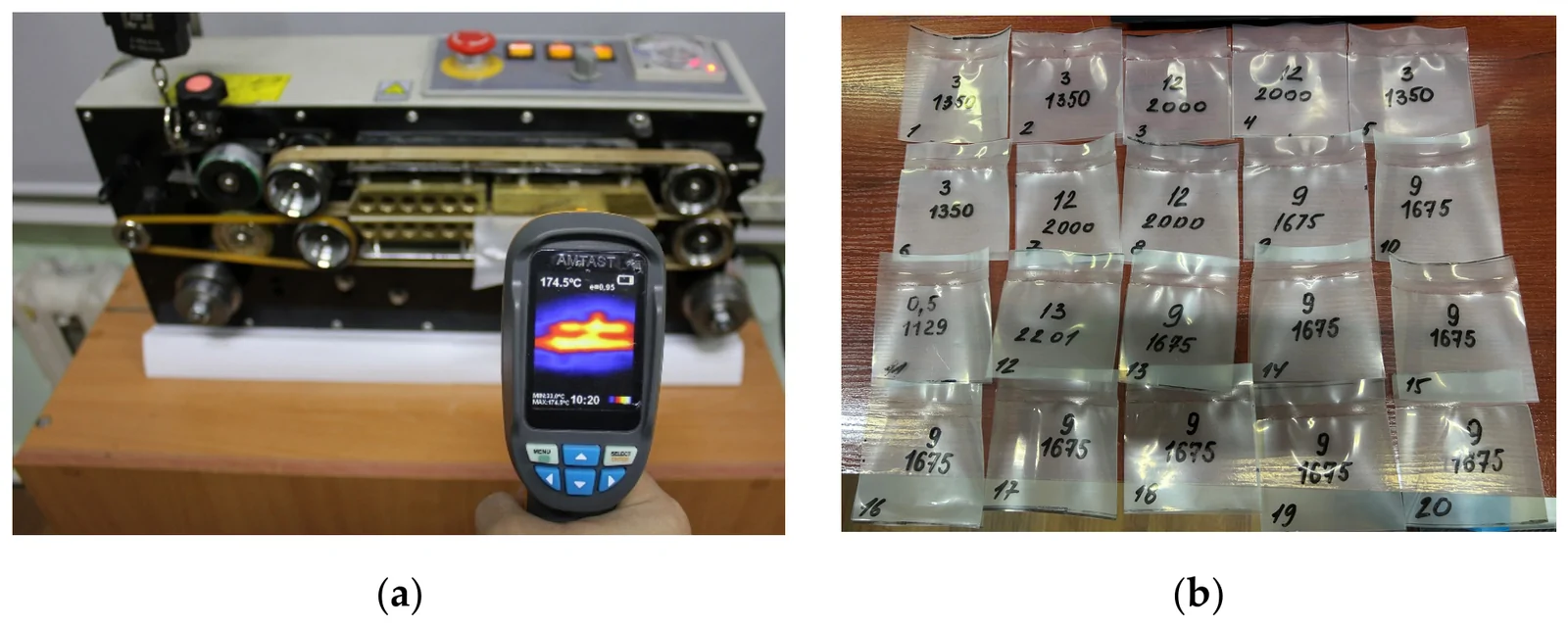
Thermal welding of polyethylene film: (**a**)—temperature control of the welding device with an Amtast AMF101 heat detector; (**b**)—samples of polyethylene film obtained under different welding conditions.

Before the start of each experimental mode, the welding device was heated to a specified temperature of 175 °C. The heating mode of the mobile welding module heater was controlled by an “Amtast AMF101” heat detector with a temperature measurement range from 20 to 300 °C. After reaching and stabilizing the specified temperature, the required compressive force and the specified weld width were set. The time of thermal exposure was taken as 3 s according to the previously developed methodology [18]. The prepared sample was delivered to the welding module’s work area in such a way that the weld formation section passed through the thermal impact and compression area. After passing through the work area, the sample was removed and left until the weld joint was stabilized to ambient temperature. The welded specimen was subsequently marked to identify the conditions of the experiment (Figure 2).

After the weld was formed, the thickness of the weld was measured using a thickness gauge from ET 11R. The values obtained were recorded for each sample and used in the subsequent analysis of the influence of the process parameters on the condition of the weld zone.

The strength of welded PE film seams was assessed by applying negative pressure.

**Figure 3:**
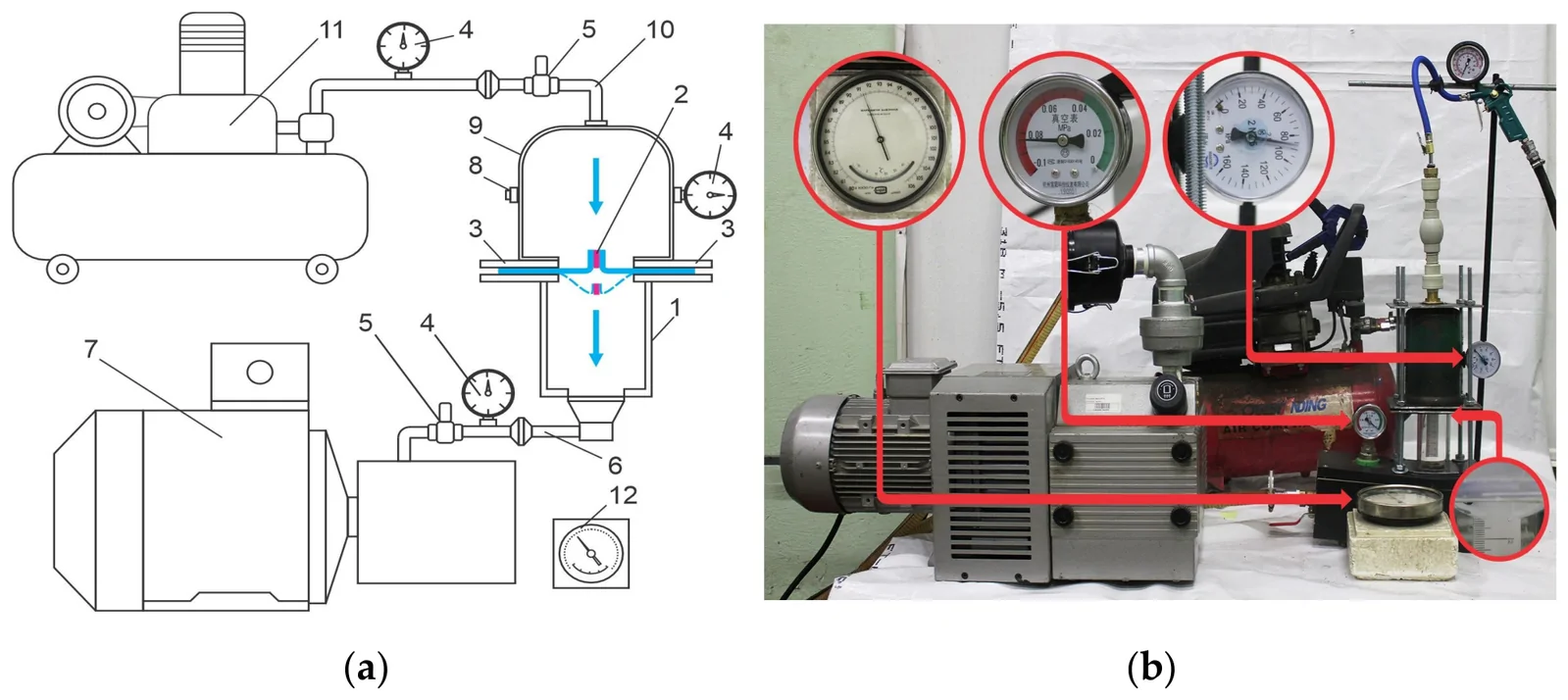
Diagram (**a**) and overall view (**b**) of weld strength test device: 1—graduated glass cylinder with open top; 2—sample polyethylene film with weld; 3—clamp; 4—vacuum gauge; 5—shut-off valve; 6—vacuum line; 7—vacuum pump; 8—control valve; 9—vessel for creating excess pressure; 10—pressure line; 11—air pump; 12—barometer.

Welded samples were resealed on the test device one at a time and then pressurized with a vacuum pump. The maximum dilution value immediately at the moment of breaking down the weld was recorded on a vacuum gauge (Figure 3).

The prepared specimen (2) is hermetically secured to the open end of the cylinder receptacle (1) using a clamping element (3). Then, by means of a vacuum pump (7), a vacuum was created inside of the container. Due to a lack of atmospheric pressure (according to the geolocation), excessive pressure was applied by means of a compressor (11) through a pressure line (10) into the vessel (9) for maintaining pressure. Pressure in the vessel was maintained by a valve (8). Under negative pressure, the film was deformed inside the cylinder into a hemispherical shape, as a result of which the weld was subjected to tensile stress. The test continued until the welded joint or adjacent film section has broken down. The value of the vacuum pressure at the moment of rupture was taken as a measure of the weld strength limit. For each welding mode, weld-break tests were performed with a 5-fold repetition required to statistically process the results. The objective of this study was to obtain a correlation relationship (burst vacuum pressure, P in MPa) with the following parameters: width of the weld seam (b in mm); clamping force of polyethylene films during welding (*F* in N). To introduce the study of the strength of welded polyethylene film compounds by a multifactor experiment, a method for planning the experiment was adopted by standard procedure [29,30,31]. The intervals between the independent variable (Table 2) and the experimental plan matrix (Table 3) are as follows.

## 3. Results

### 3.1. Numerical Results

**Figure 4:**
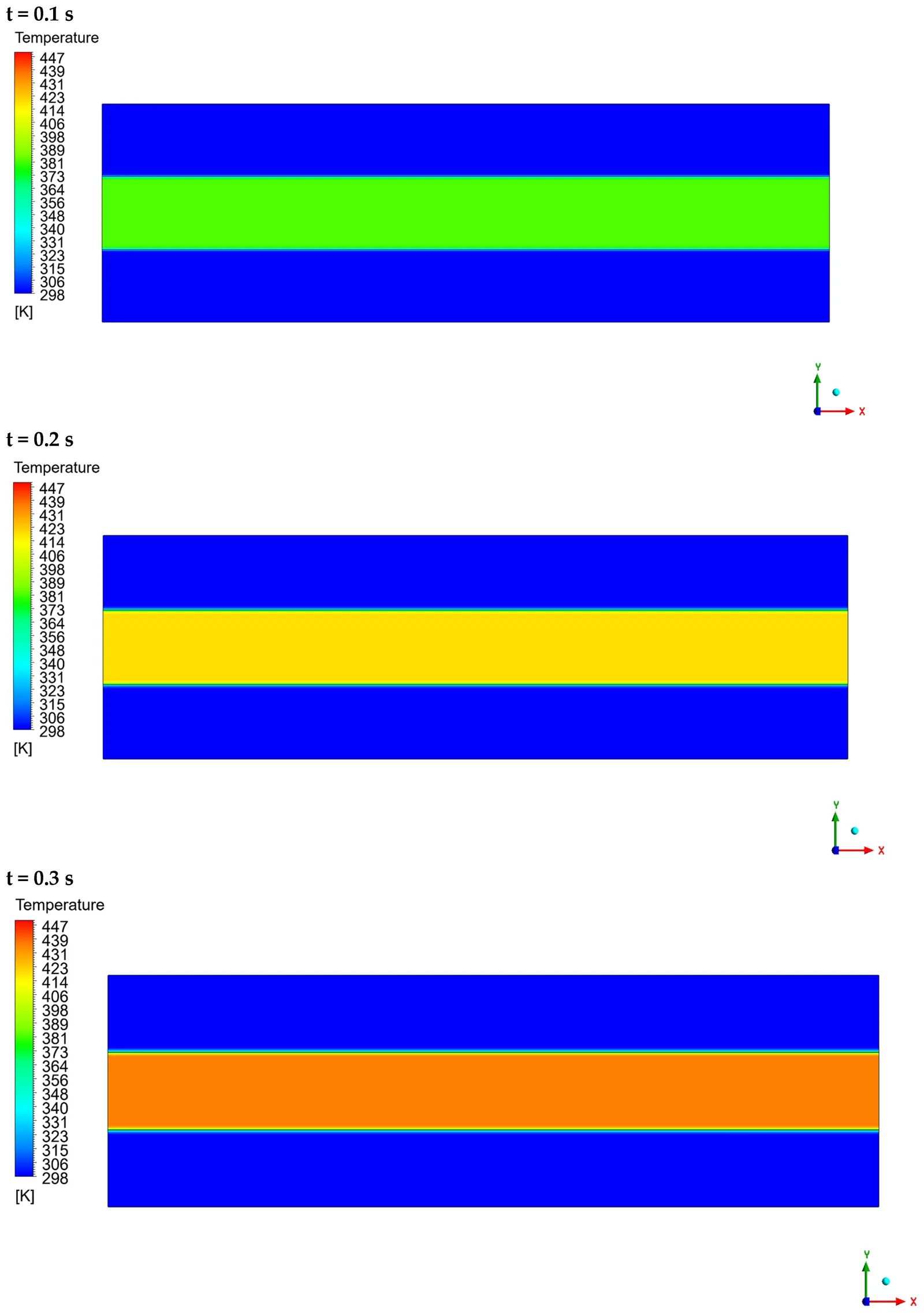

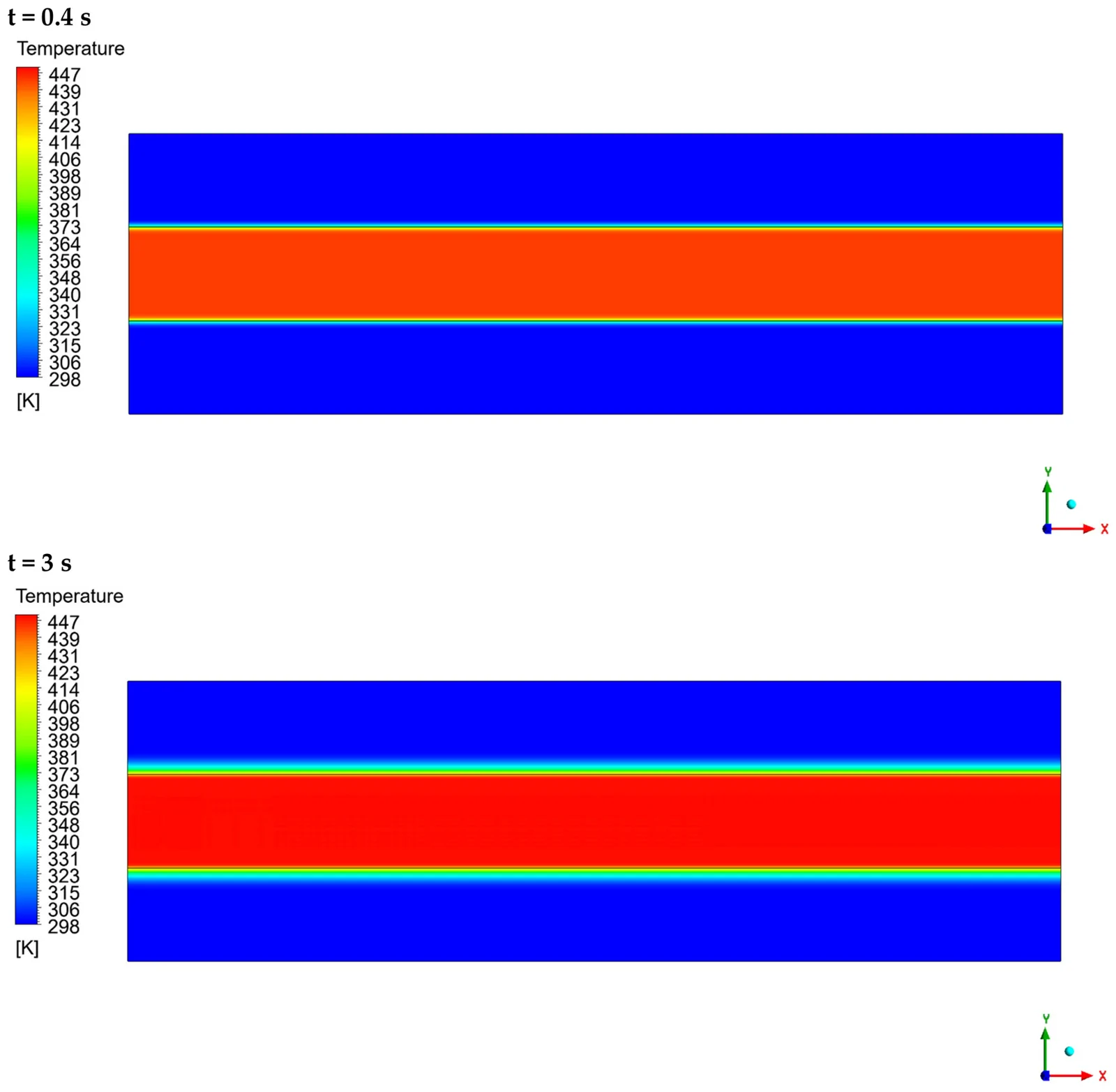
Temperature contours at different time instants (on horizontal plane).

**Figure 5:**
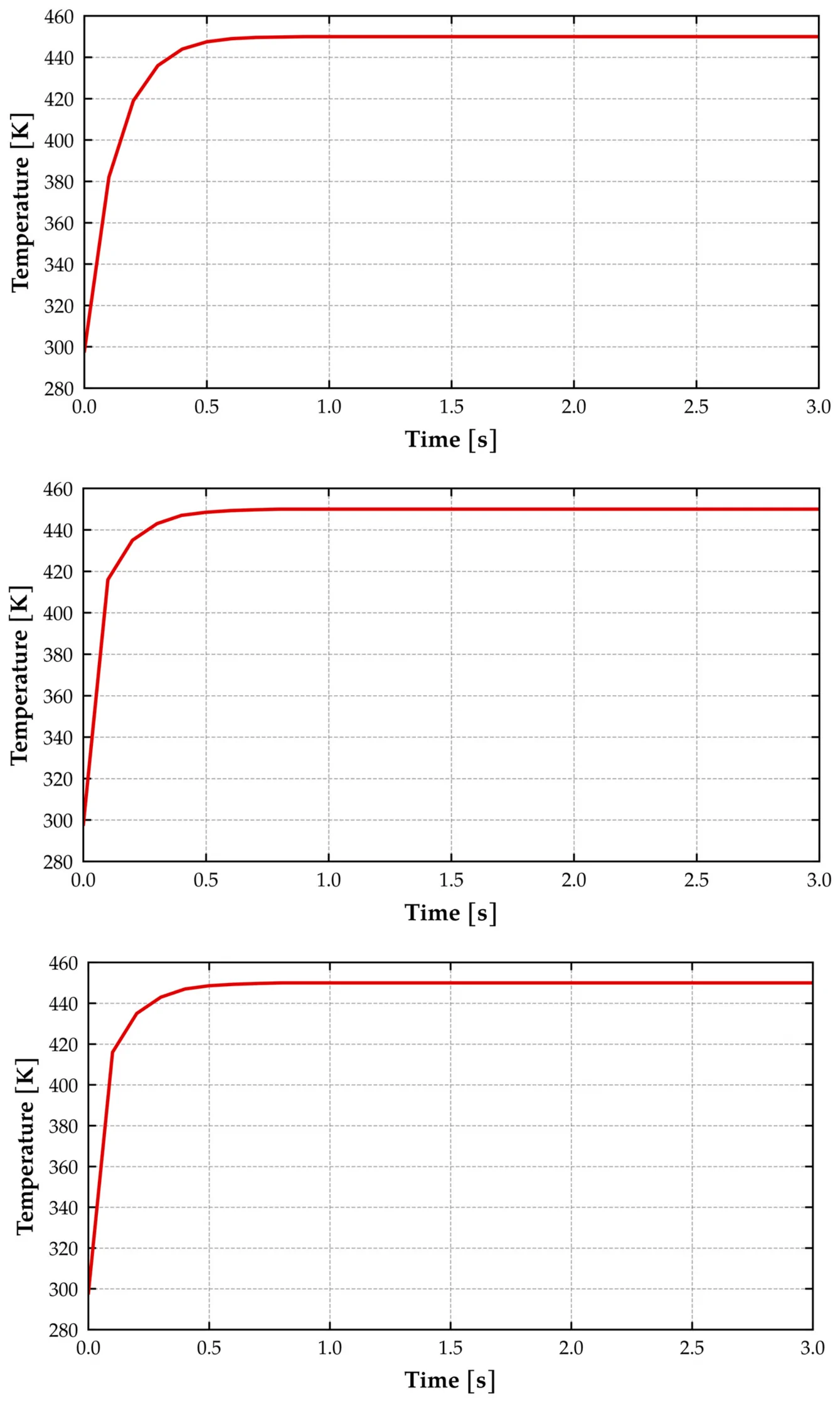
Temperature–time curves at points *P*_1_, *P*_2_ and *P*_3_, respectively.

Figure 4 shows temperature contours at different time instants; heating occurs along a thin horizontal line (surface of a weld—top view):

To analyze the dynamics of material heating during the welding process, the temperature history *T*(*t*) was examined at fixed points *P*_1_, *P*_2_, *P*_3_ with the following coordinates (Table 4):

Temperature–time curves at points P_1_, P_2_ and P_3_, respectively shown on Figure 5.

**Figure 6:**
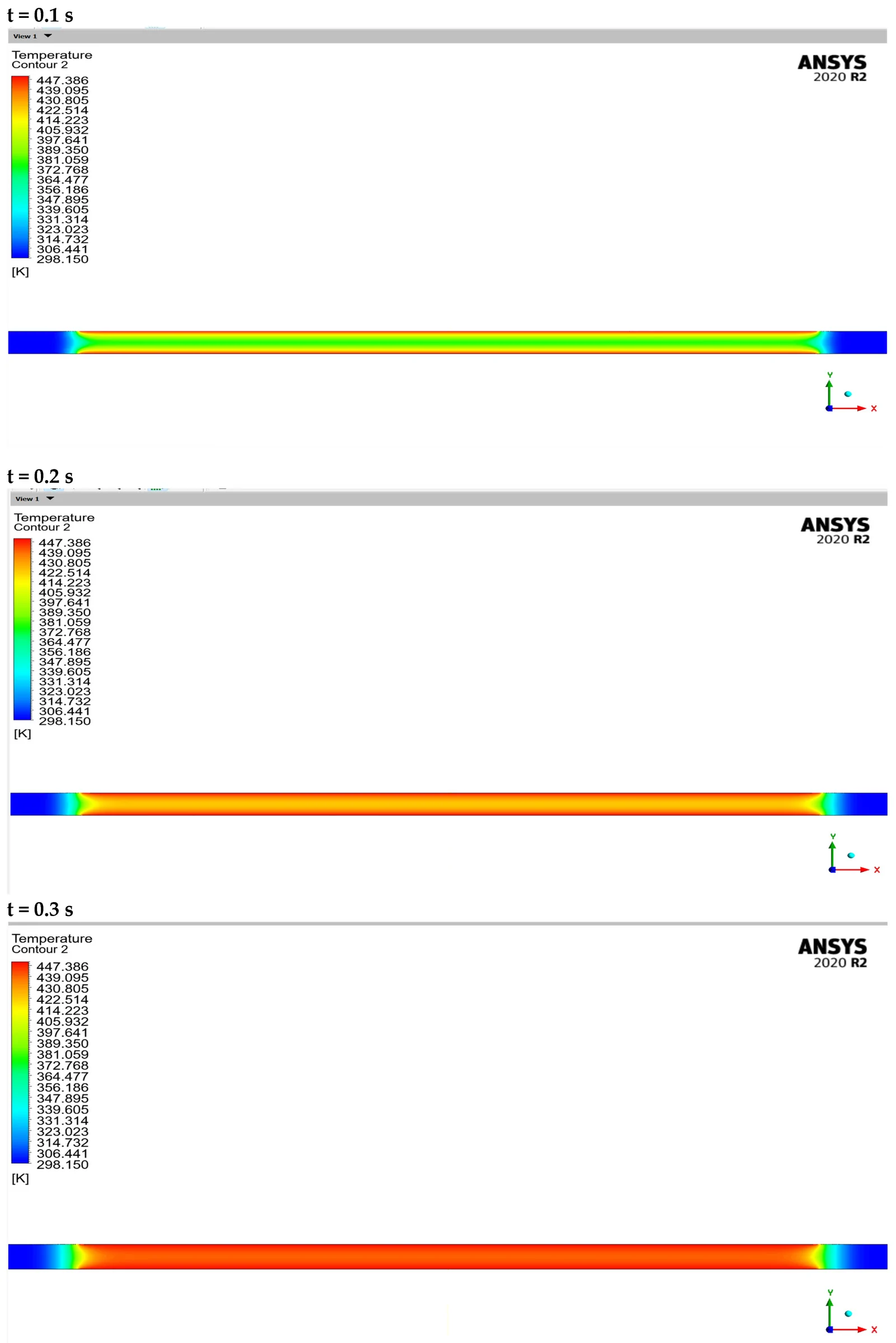

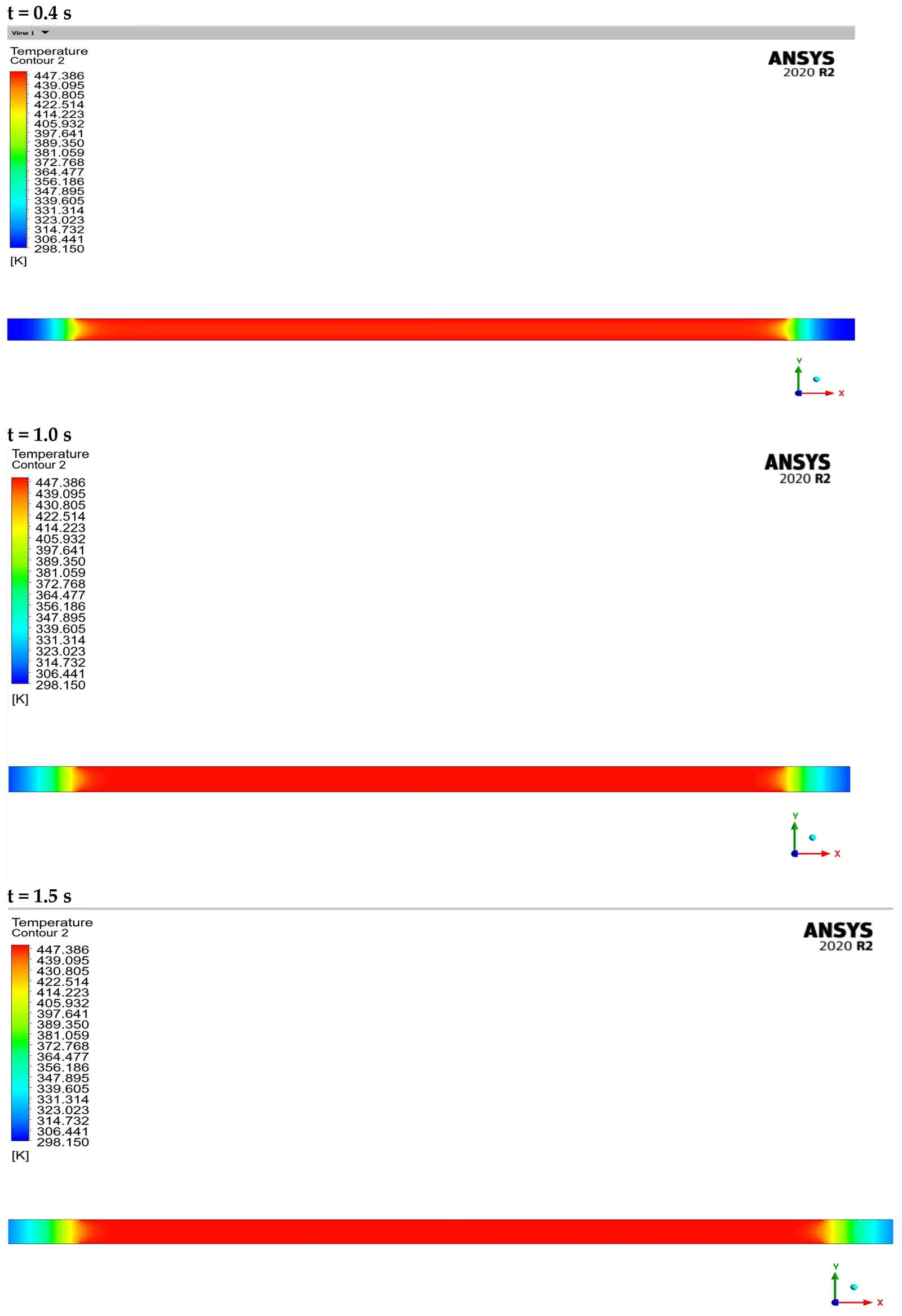
Temperature contours at different time instants.

**Figure 7:**
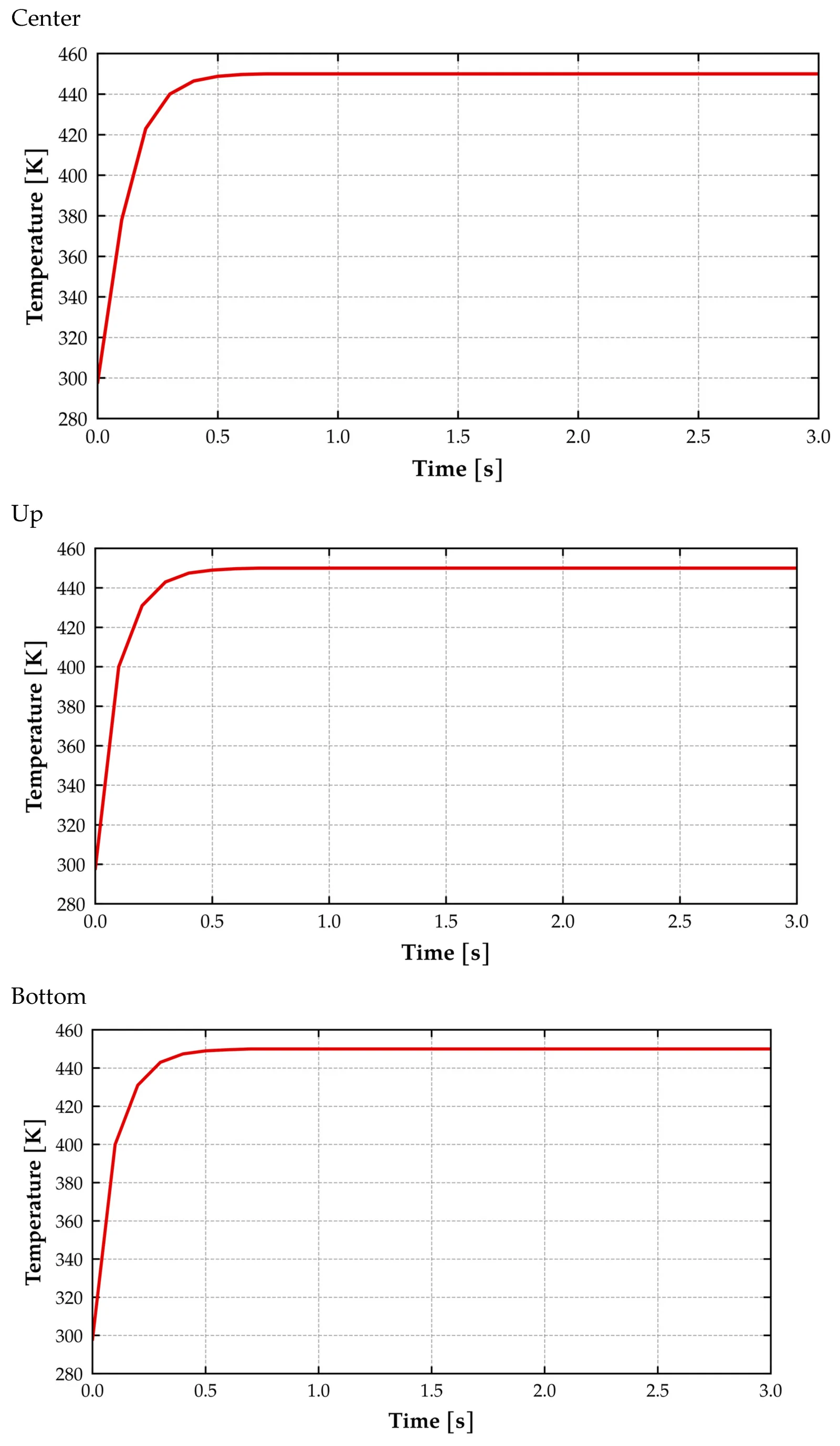
Temperature–time curves at points *P*_1_, *P*_2_ and *P*_3_, respectively.

Figure 6 shows temperature contours at different points in time; heating occurs from above for the upper film and from below for the lower film (cross-section of the weld seam along the longitudinal surface—side view):

In a similar analysis of the dynamics of material heating during the welding process, the temperature history *T*(*t*) was examined on the Figure 7 at fixed points *P*_1_, *P*_2_, *P*_3_ with the following coordinates (Table 4):

### 3.2. Experimental Results

The experimental data processing found that the estimated surface response values by vacuum burst pressure in the range studied varied from 0.080 to approximately 0.094 MPa. The resulting dependency is described by a second-order regression equation:

(2) $$Y=84,139+3228X_{1}-4928X_{2}+6125X_{1}X_{2}+2839X_{1}^{2}+0.182X_{2}^{2}.$$

To construct the response surface, a quadratic regression equation in natural variables is used.

(3) *P* = 226,356 − 22,717*b* − 8072*F* + 0.960*bF* + 0.714*b*^2^ + 0.0233*F*^2^,

where *b*—weld stitch width, mm; *F*—force exerting pressure on the film being welded, N.

**Figure 8:**
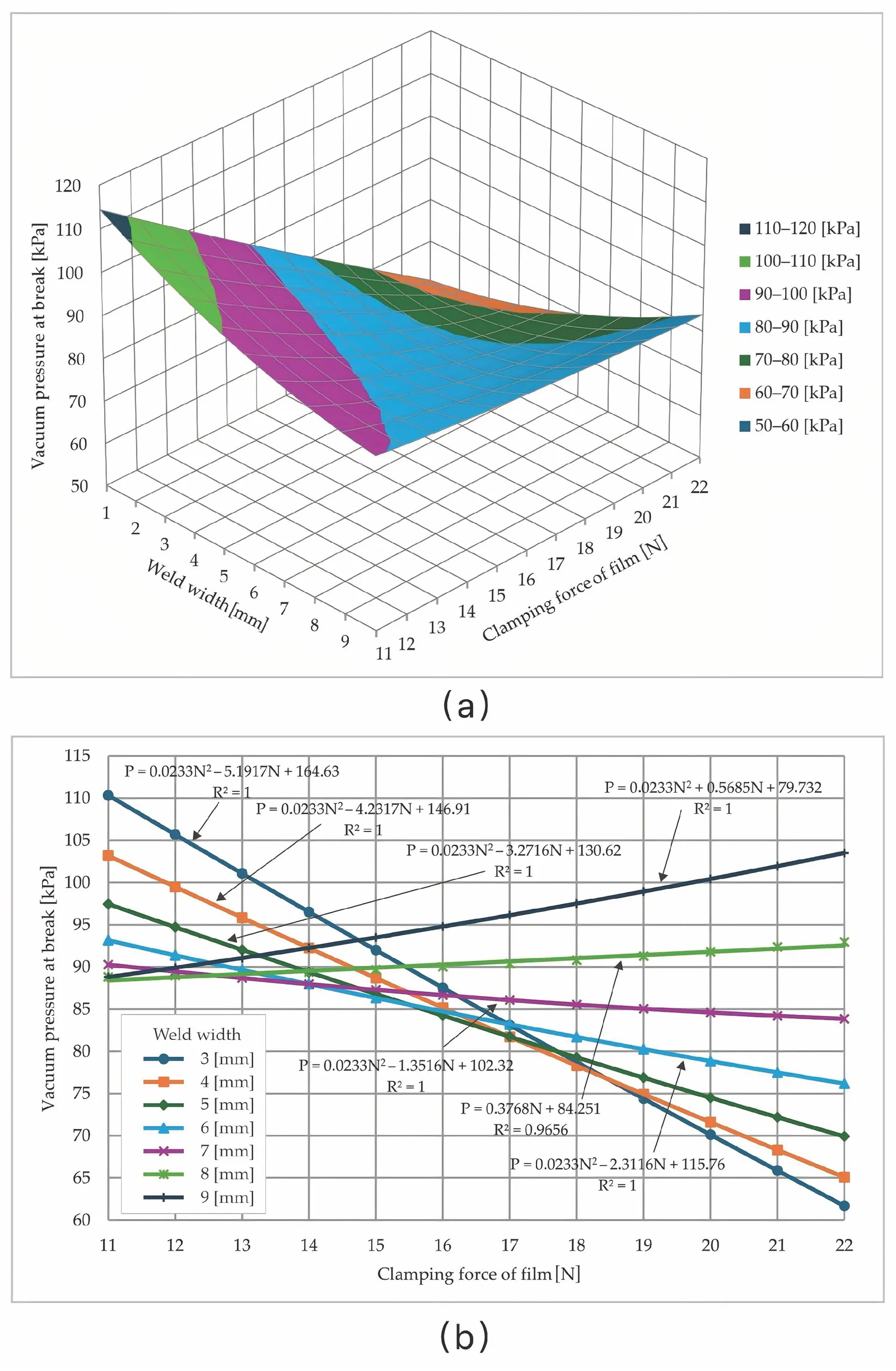
Dependence of the vacuum-breaking pressure of the film joints on the width of the joint and the welding pressure (**a**) 3D and (**b**) 2D.

The graphical interpretation of the resulting dependence in the form of a three-dimensional response surface shows how the weld strength changes smoothly over the entire range studied in (Figure 8).

**Figure 9:**
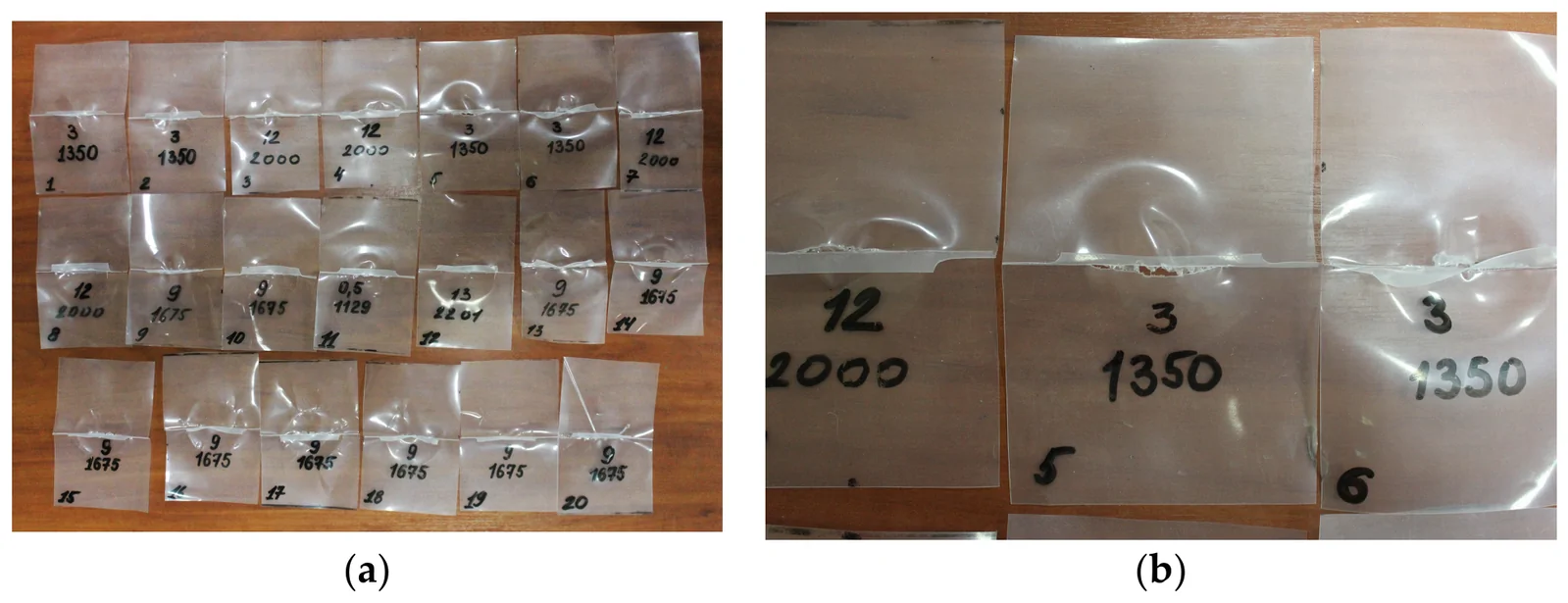
The breaking status of samples tested: (**a**) one from each repetition and (**b**) a zoomed-in view of the break location.

Figure 9 shows the breaking status of samples (from each repetition is one) tested.

## 4. Discussion

The reliability of welded joints in thin polyethylene films is particularly important for flexible containers used in the vacuum packaging and preservation of succulent forage, including alfalfa haylage. During vacuum compaction, the container walls and welded seams are subjected to mechanical stresses resulting from the pressure difference between the internal and external environments. The reliability of these joints depends on several technological parameters, particularly sealing temperature, weld-seam width, and clamping force.

In thin polyethylene films, excessive heating may cause substantial softening and displacement of polymer material, resulting in localized thinning of the welded region. Excessive clamping force may further promote material displacement and reduce the effective thickness of the joint. Conversely, insufficient clamping force may prevent intimate contact between the softened polymer surfaces, thereby limiting the formation of a continuous welded interface. Consequently, controlling the thermal conditions and mechanical compression during welding is essential for achieving reliable joints.

In our previous study [18], an analytical heat-conduction approach was used to determine suitable temperature conditions for polyethylene film welding. In the present investigation, three-dimensional transient numerical modeling was employed to obtain a more detailed understanding of heat propagation and temperature distribution within the polyethylene film during the sealing process.

The numerical results demonstrate that the heating behavior strongly depends on the distance from the heating-element axis. At the monitoring point located directly beneath the center of the heater, the temperature increased rapidly during the initial 0.1 s, after which the heating rate gradually decreased. After 3 s, the temperature reached approximately 442 K, approaching the prescribed heating-element temperature of 450.15 K. At monitoring points located 5 and 9 mm from the heater axis, the corresponding temperatures were approximately 376 and 317 K, respectively. These results indicate that the thermal effect decreases considerably with increasing lateral distance from the heating source.

The differences in temperature between the monitoring points demonstrate the localized nature of heat transfer during welding. The most intensive heating occurs in the immediate vicinity of the heating-element axis, while the temperature rise becomes substantially smaller at greater distances. This temperature distribution provides a basis for estimating the effective width of the heat-affected region and evaluating the thermal conditions required for welded-joint formation.

The experimental results were analyzed using a second-order regression model to evaluate the combined effects of weld-seam width and clamping force on the rupture pressure of polyethylene film joints. The positive linear coefficient associated with weld-seam width (b_1_) indicates a tendency for rupture pressure to increase with increasing seam width when the other model terms are taken into account. In contrast, the negative linear coefficient associated with clamping force (b_2_) indicates a negative contribution of this factor to the predicted response at the reference level of the other factor. However, these individual effects must be interpreted in conjunction with the interaction and quadratic terms included in the model.

The positive and statistically significant interaction coefficient (b_12_) indicates that the influence of weld-seam width on rupture pressure depends on the applied clamping force, and vice versa. The statistically significant quadratic term X_1_^2^ further indicates a nonlinear relationship between seam width and rupture pressure. In contrast, the quadratic term X_2_^2^ was not statistically significant, suggesting that the available experimental data do not provide sufficient evidence of curvature associated with clamping force alone.

The partial correlation coefficients calculated for the individual model terms, while controlling for the remaining terms, indicated relatively strong associations for clamping force (X_2_, −0.719) and the interaction between seam width and clamping force (X_1_X_2_, 0.701). The partial correlation associated with X_2_^2^ was close to zero, consistent with its statistically insignificant contribution to the fitted model.

The coefficient of determination (R^2^ = 0.7417) indicates that the quadratic regression model explained approximately 74.2% of the observed variability in rupture pressure. The multiple correlation coefficient (R = 0.8612) indicates a positive correlation between the measured and predicted response values. The mean relative approximation error was 3.589%, suggesting a relatively small average discrepancy between the experimental and calculated values. Nevertheless, this aggregate measure does not exclude systematic deviations within particular regions of the experimental domain.

The three-dimensional response surface (Figure 8) illustrates the combined influence of weld-seam width and clamping force on the predicted rupture pressure. Within the investigated parameter range, the modeled response varied approximately from 0.080 to 0.099 MPa. The response surface indicates that certain combinations of the investigated factors are associated with higher rupture pressures. However, these combinations should be regarded as preliminary favorable operating conditions rather than fully validated optimal welding parameters.

The physical interpretation of these relationships is associated with the conditions governing welded-joint formation. Increasing seam width may enlarge the effective bonded area and improve resistance to mechanical loading. Adequate clamping force promotes contact between the softened polyethylene surfaces and facilitates joint formation. However, excessive compression may displace softened polymer material, causing localized thinning and potentially reducing the resistance of the welded region to rupture.

An important experimental observation was that failure occurred in the polyethylene film adjacent to the welded seam, while the seam itself remained intact. This failure mode indicates that, under the investigated testing conditions, the welded joints were sufficiently strong to withstand the applied pressure difference without direct separation along the welded interface. Nevertheless, localized thinning in the heat-affected region may influence the overall failure resistance of the specimen. These observations support the practical potential of the investigated heat-sealing parameters for producing reliable joints in 200 μm polyethylene film.

Several limitations should be considered when interpreting the results. The numerical model was developed using prescribed thermophysical properties and heating conditions, while the experimental investigation was performed at a constant sealing temperature. Furthermore, the statistically significant lack of fit indicates that the quadratic regression model does not fully capture the experimental response. Therefore, additional experimental validation and refinement of the model are required before its application to quantitative optimization or reliable prediction outside the tested conditions.

Future investigations should focus on improving the predictive accuracy of the regression model and evaluating the long-term reliability of welded polyethylene film joints under practical storage conditions. Particular attention should be given to environmental influences, including solar radiation and low temperatures, which may affect the durability and sealing performance of flexible polymer containers [22,32].

## 5. Conclusions

This study investigated the reliability of heat-sealed joints in thin polyethylene films used for flexible containers intended for vacuum compaction and storage of succulent forage. A combined numerical and experimental approach was employed to investigate transient heat transfer during welding and evaluate the effects of weld-seam width and clamping force on resistance to pressure-induced rupture. The main findings are summarized as follows:

1. A three-dimensional transient heat-conduction model was developed to investigate temperature distributions in polyethylene film during heat sealing. The numerical results demonstrated rapid localized heating and a decrease in temperature with increasing distance from the heating element. The model provides a basis for estimating the extent of the heat-affected region and evaluating thermal conditions during welded-joint formation. However, it does not account for phase transitions, molten polymer flow, or macromolecular interdiffusion across the welded interface.
2. Experimental investigations established the combined influence of weld-seam width and clamping force on the rupture pressure of welded polyethylene film specimens. Within the investigated range of welding parameters, the measured rupture pressure varied from 0.080 to 0.094 MPa. Visual examination of all 100 tested specimens showed that rupture occurred in the polyethylene film adjacent to the welded region, while the welded seams remained intact. This observation demonstrates the resistance of the welded interfaces to separation under the investigated loading conditions.
3. A second-order polynomial regression model was developed to describe the relationship between rupture pressure and the investigated welding parameters. Analysis of variance indicated statistically significant effects of weld-seam width, clamping force, and their interaction. The model explained approximately 74.2% of the observed variation in rupture pressure (R^2^ = 0.742). However, the statistically significant lack of fit indicates that further refinement and experimental validation are required before the model can be used for accurate quantitative prediction and optimization.
4. The combined numerical and experimental findings provide a basis for selecting heat-sealing parameters to improve the reliability of flexible polyethylene containers used for forage preservation. Further research should focus on incorporating temperature-dependent thermophysical properties, phase transitions, and molten polymer flow into the numerical model, as well as evaluating the long-term durability of welded joints under environmental influences, including temperature variations and solar radiation.

## Figures and Tables

**Table 1:** The thermophysical properties of polyethylene for calculation.

Property	Symbol	Value	Unit
Density	*ρ*	955	kg/m^3^
Specific heat capacity	*C_p_*	2250	J/(kg·K)
Thermal conductivity	*k*	0.54	W/(m·K)

**Table 2:** Value of factor levels and intervals.

Marking	Code Value	Factors	
		Weld Width, mm	Clamping Force of the Films During Welding, N
Basic level	0	6	16.42
Range of variation	ε	2	3.18
Upper level	+1	8	19.61
Lower level	−1	4	13.23
Upper star point	+1.68	9.36	21.58
Lower star point	−1.68	2.64	11.07
Code mark		X_1_	X_2_

**Table 3:** Plan matrix and factor variation levels.

Experiment Numbers	Weld Width		Clamping Force of Film		Average Vacuum Pressure Values at the Moment of Rupture
	*X*_1_	*b*, mm	*X*_2_	*F*, N	*P_av_*, MPa
1	−1	4	−1	13.23	93.6
2	+1	8	−1	13.23	93.6
3	−1	4	+1	19.61	74.6
4	+1	8	+1	19.61	99.6
5	−1	4	−1	13.23	99.6
6	+1	8	−1	13.23	94.6
7	−1	4	+1	19.61	75.6
8	+1	8	+1	19.61	94.6
9	−1.68	2.64	0	16,42	85.6
10	+1.68	9.36	0	16.42	88.6
11	0	6	−1.68	11.07	88.6
12	0	6	+1.68	21.58	70.6
13	0	6	0	16.42	81.6
14	0	6	0	16.42	83.6
15	0	6	0	16.42	83.6
16	0	6	0	16.42	85.6
17	0	6	0	16.42	82.6
18	0	6	0	16.42	82.6
19	0	6	0	16.42	83.6
20	0	6	0	16.42	81.6

**Table 4:** Coordinates of fixed points.

Coordinate Designations	Coordinates of the Monitoring Points		
	*x*	*y*	*z*
*P*_1_	0.05	0.015	0.0002
*P*_2_	0.05	0.015	0.0001
*P*_3_	0.05	0.015	0.0003

## Data Availability

The original contributions presented in this study are included in the article. Further inquiries can be directed to the corresponding author.

## Abbreviations

The following abbreviations are used in this manuscript:

Latin Symbols	
b	Weld stitch width
*C_p_*	The specific heat capacity
*T*(*x*,*y*,*z*,*t*)	Temperature
F	Force
P	Pressure
*R*	The multiple correlation coefficient
*R*^2^	Multiple determinism coefficient
*k*	The thermal conductivity
*Gr*	Error of the multiple correlation coefficient
Greek Symbols	
$\alpha =\frac{k}{\rho C_{p}}$	The thermal diffusivity
*ρ*	The material density
Units	
K	Kelvin
kg/m^3^	Kilograms per cubic meter
J/(kg·K)	Joules per kilogram per kelvin
W/(m·K)	Watts per meter per kelvin
m	Meter
m^3^	Cubic meter
mm	Millimeter
s	Second
Pa	Pascal
MPa	Megapascal
kPa	Kilopascal
N	Newton
°C	Degree Celsius
Acronyms	
ANSYS	Analysis systems
PE	Polyethylene
LDPE	Low-Density Polyethylene
HDPE	High-Density Polyethylene
